# Structural basis for pathogenic variants of GJB2 and hearing levels of patients with hearing loss

**DOI:** 10.1186/s13104-024-06793-w

**Published:** 2024-05-10

**Authors:** Kazunori Namba, Hideki Mutai, Tatsuo Matsunaga, Hiroki Kaneko

**Affiliations:** 1 Division of Hearing and Balance Research, National Institute of Sensory Organs, NHO Tokyo Medical Center, 2-5-1 Higashigaoka, Meguro-ku, Tokyo, 152-8902 Japan; 2 Medical Genetics Center, NHO Tokyo Medical Center, 2-5-1 Higashigaoka, Meguro-ku, Tokyo, 152-8902 Japan; 3 Health and Nutrition (NIBIOHN), National Institutes of Biomedical Innovation, 7-6-8 Saito-Asagi, Ibaraki, Osaka 567-0085 Japan; 4 The Institute of Natural Sciences, College of Humanities and Sciences, Nihon University, 3-25-40 Sakurajousui, Setagaya-ku, Tokyo, 156-8550 Japan

**Keywords:** Hereditary hearing loss, GJB2, Gap junction, Connexin, Molecular modeling, Genotype–phenotype correlation

## Abstract

**Objectives:**

The crystal structure of the six protomers of gap junction protein beta 2 (GJB2) enables prediction of the effect(s) of an amino acid substitution, thereby facilitating investigation of molecular pathogenesis of missense variants of *GJB2*. This study mainly focused on R143W variant that causes hearing loss, and investigated the relationship between amino acid substitution and 3-D structural changes in GJB2.

**Methods:**

Patients with nonsyndromic hearing loss who appeared to have two *GJB2* pathogenic variants, including the R143W variant, were investigated. Because the X-ray crystal structure of the six protomers of the GJB2 protein is known, R143W and structurally related variants of GJB2 were modeled using this crystal structure as a template. The wild-type crystal structure and the variant computer-aided model were observed and the differences in molecular interactions within the two were analyzed.

**Results:**

The predicted structure demonstrated that the hydrogen bond between R143 and N206 was important for the stability of the protomer structure. From this prediction, R143W related N206S and N206T variants showed loss of the hydrogen bond.

**Conclusion:**

Investigation of the genotypes and clinical data in patients carrying the R143W variant on an allele indicated that severity of hearing loss depends largely on the levels of dysfunction of the pathogenic variant on the allele, whereas a patient with the homozygous R143W variant demonstrated profound hearing loss. We concluded that these hearing impairments may be due to destabilization of the protomer structure of GJB2 caused by the R143W variant.

**Supplementary Information:**

The online version contains supplementary material available at 10.1186/s13104-024-06793-w.

## Introduction

*GJB2* is the most frequent causative gene of autosomal recessive nonsyndromic sensorineural hearing loss (DFNB1A, OMIM: 220290) worldwide [1, 2]. *GJB2* encodes connexin 26, which forms a hemichannel or connexon composed of six protomers (subunits), enabling the exchange of ions and small molecules between cells. A gap junction that allows the transport of small molecules, including K + , in the cochlear cells and is believed to be essential to maintaining high K + concentration in the cochlear endolymph via ion-recycling in the cochlea [3]. Normal ion exchange occurs when the gap junction formed by two connexons achieve the correct three-dimensional (3-D) structure.

Hearing levels of the patients having biallelic missense variants of *GJB2* vary from mild to profound [4–6]. This could be attributed to the extent of the level of dysfunction of the GJB2 missense variants, based on the fact that a compound heterozygote with two nonsense or truncated variants generally leads to more severe phenotypes [4, 5]. Various studies have attempted to elucidate the extent of molecular pathology of GJB2 variants via in vitro analyses [7–9]. However, due to technical differences, the molecular pathology of some variants remains controversial [7–9], even though the pathogenicity of variant which change an arginine at position 143 of the GJB2 to a tryptophan (R143W) (NM_004004.6:c.427C > T) has been established clinically [10, 11].

In Ghana, R143W is the largest contributor to non-syndromic hearing impairment and has a reported prevalence of 25.9% in affected multiplex families [12].

To understand the molecular pathology of each missense variant of *GJB2*, predicting the structural changes in GJB2 caused by each variant is considered an alternative strategy, as it provides visual information of the structural change at the atomic level. In addition, it could provide insights for designing specific drugs to attenuate or block the dysfunctional functioning by changing the residues.

The crystal structure of the gap junction channel revealed two membrane-spanning hemichannels consisting of six GJB2 protomers [13]. Notably, R143 has been shown to interact with N206 through a hydrogen bond [13]. The two residues reside in adjacent transmembrane regions (helix-3 and helix-4) and are considered to contribute to the stability of the protomer. Interestingly, not only the R143W but also the N206S (c.617A > G) [14] and N206T (c.617A > C) [15] variants have been reported to be associated with hearing loss.

This study was aimed at predicting the structural changes in GJB2 caused by the R143W as well as the N206S and N206T variants and investigating whether the hydrogen bond between the two residues was affected. We also assessed whether the 3-D structure of GJB2 was altered by the V37I variant. Patients carrying the R143W variant in one allele and a pathogenic variant of *GJB2* in the other were included, and the correlation between the genotypes and hearing levels of patients was investigated.

## Methods

### Molecular modeling of GJB2 variants

The R143W, N206T, N206S, and V37I variants of GJB2 were modeled on SWISS-MODEL [16, 17] using the crystal structure of GJB2 (PDB: 2zw3, A chain) [13] as a template. SWISS-MODEL is a fully automated protein homology-modeling server and one of the most widely used tools with high reliability [18]. In our previous study, the structures of its models showed high scores using the structural evaluation software Verify 3D [19–21]. Furthermore, to confirm the accuracy of the models, the 3-D structures corresponding to each model were predicted using Alphafold2 [22, 23], which is a novel and powerful machine learning approach with the highest reliability among the options currently available. The calculations were carried out in ColabFold v1.5.5: AlphaFold2 using MMseqs2 [24]. All diagrams were created using UCSF Chimera [25] to visualize ribbon models with the hydrogen bonds.

### Subjects

Genetic testing for *GJB2* variants by direct sequencing was performed on 74 individuals (46 families) with hearing loss, and 42 patients (37 families) were found to carry biallelic *GJB2* variants. In total, 22 patients with nonsyndromic hearing loss who appeared to have two *GJB2* pathogenic variants including at least one R143W variant were investigated. Hearing levels were examined using pure-tone audiometry, auditory steady-state response, or conditioned orientation reflex audiometry, according to the age of the patients. Hearing levels of those with better hearing were classified according to the recommendations of the Genetic Deafness study group [26]. Based on our flowchart, genetic analyses of *GJB2* were performed on patients with nonsyndromic hearing loss in which nongenetic causes were excluded [27]. To predict the pathogenicity of each variant, the REVEL scoring system, an in silico pathogenicity predictor of missense variants [28], was used. Scores ≥ 0.7 were considered to be indicative of pathogenicity [29].

## Results

**Fig. 1.**
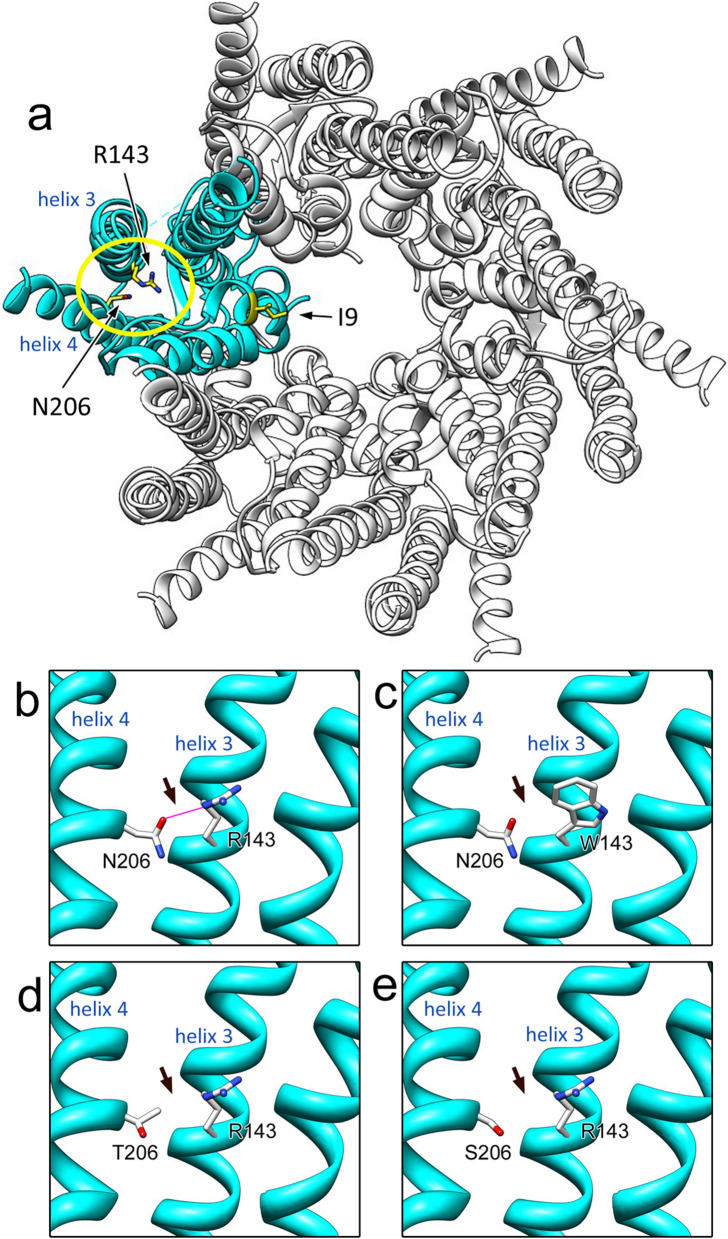
3-D structures of the wild-type and variants of GJB2. **a** The hemichannel crystal structure consisting of six wild-type GJB2 proteins. The positions R143 and N206 in one of the GJB2 hexamers are indicated by yellow circles. **b** Hydrogen bond between R143 on helix 3 and N206 on helix 4 of wild-type proteins is indicated in red. **c**–**e** The hydrogen bond was absent in R143W (**c**), N206T (**d**), and N206S (**e**) variants. Arrows indicate the position of the hydrogen bonds

The crystal structure of GJB2 was used as a template; then, a structural model for the R143W variant was constructed, and the resulting mechanisms of dysfunction from the viewpoint of structural biology were investigated. R143 was located in helix 3 of GJB2, and by virtue of its hydrogen bonding to N206 in helix 4, stability of the subunit structure (one unit of six protomers) was thought to be maintained (Fig. 1a, b). The hydrogen bond disappeared after substituting R143 with tryptophan (W), as shown in our structural model (Fig. 1c). Moreover, N206T and N206S have both been reported as pathogenic variants of GJB2 [14, 15, 30], which are variants of the corresponding partner residue, i.e., N206 for hydrogen bonding with R143. The REVEL scores [28] predicted the R143W as well as the N206T and N206S variants to be pathogenic (R143W; 0.918, N206T; 0.826, N206S; 0.775), supporting the findings of previous reports. Prediction of the 3-D structural models of the N206T and N206S variants revealed absence of the hydrogen bond between the side chains of the R143 and the -OH group of either the substituted T206 or S206 (Fig. 1d, e).

Structural change in another pathogenic GJB2 variant, V37I [31], was also predicted and compared with those in the R143W, N206T, or N206S variants. When patients carried the V37I variant on an allele with the R143W or N206S variant on the other allele, they showed moderate to severe hearing loss (Additional Table S1). In the structure of GJB2, the valine at position 37 is located at the center of the pore (Additional Fig. S1). By comparing the 3-D structures of the wild and variant models, it was suggested that when the valine at position 37 was substituted with isoleucine, although isoleucine has a slightly larger side chain, no significant change was observed in the hydrophobicity of the surroundings for both wild-type and variant residues (Additional Fig. S1).

**Fig. 2:**
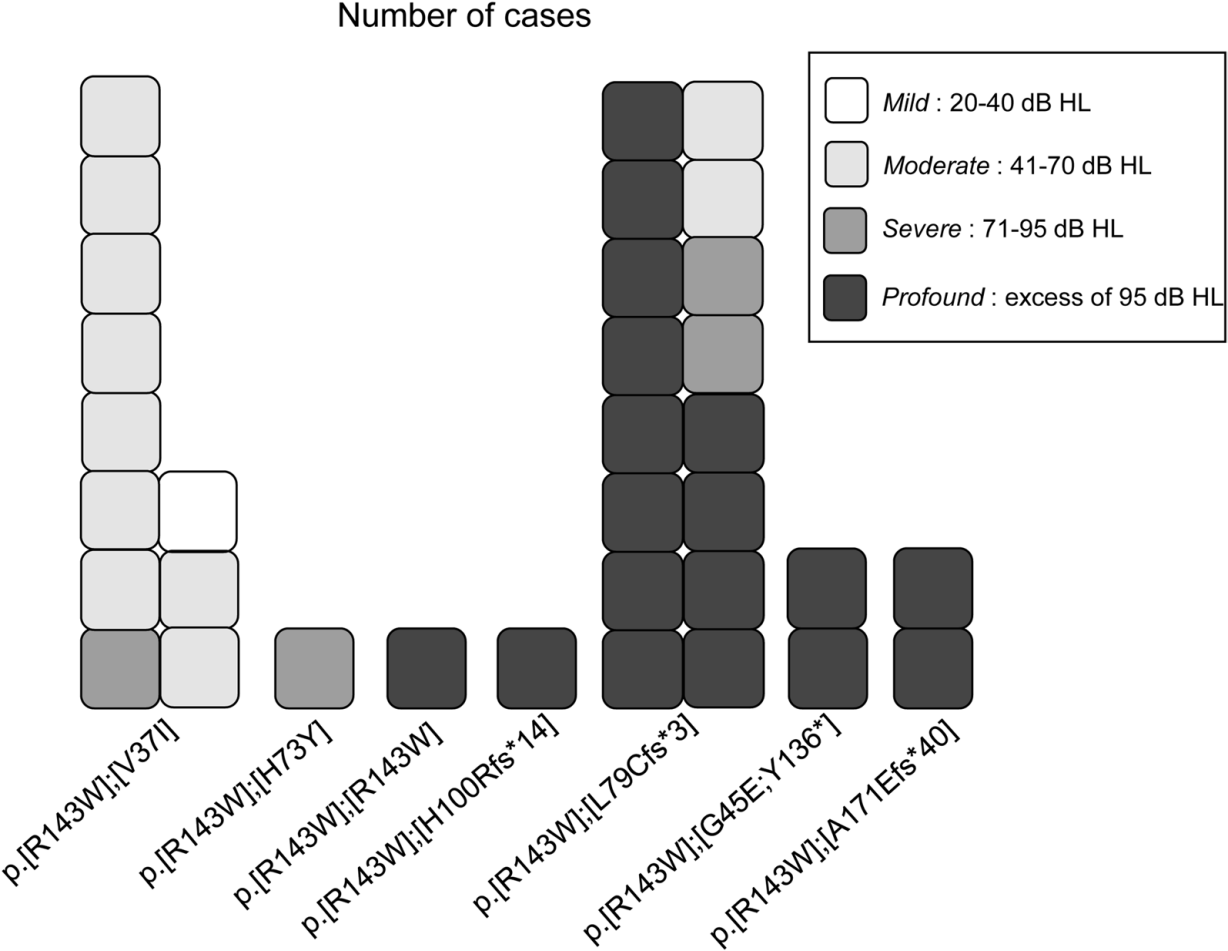
Hearing levels of patients carrying p.R143W variant of *GJB2*. Hearing levels of 22 patients with each genotype, including at least one R143W variant, were shown. See [18] for the classification of the hearing levels

Furthermore, the hearing levels of the 34 patients carrying at least the R143W variant on an allele with known pathogenic variants on the other allele were investigated (Fig. 2). The clinical data of patients with each genotype are presented in Additional Table S1. Genotype–phenotype correlations revealed that when the other allele of R143W was a missense variant among the 11 patients harboring p.[R143W];[V37I] genotype, one patient had mild hearing loss, nine patients had moderate hearing loss, whereas one had severe hearing loss. The patient with p.[R143W];[H73Y] genotype had severe hearing loss. Among the five cases having profound hearing loss, only one case had p.[R143W];[R143W] genotype. Conversely, when the other allele included a truncated variant (frameshift or nonsense variant), profound hearing loss was observed, i.e., among 21 cases having either p.L79Cfs*3(c.235delC rs80338943), p.G45E;Y136*(rs786204690), p.H100Rfs*14 (c.299_300del, rs111033204), or p.A171Efs*40 (c.508_511dup, rs773528125) on the allele other than the R143W allele, 17 had profound hearing loss (Fig. 2). Similar to those with the R143W allele, patients with p.[N206S];[V37I] genotype showed moderate hearing loss, whereas those with p.[N206S];[truncated variants] genotypes tended to show severer hearing loss (Additional Table S1).

## Discussion

This study focused on the hydrogen bond between R143 and N206 of the adjacent helices in the GJB2 subunit (one unit of six protomers) and examined its significance. Based on the crystal structure [13] and variant models, including the pathogenic variants of R143W, N206T [15], and N206S [14, 32], the hydrogen bond was absent in all cases. This finding strongly suggests that this hydrogen bond is an indispensable component in maintaining the transmembrane domain structure of the GJB2 protomer and is essential for its proper function [33]. We also built other molecular models for three variants—namely, R143W, N206T, and N206S—using Alphafold2. As a result, it became clear that the hydrogen bonding between R143 and N206 in the wild type disappeared in all three variants, indicating the accuracy of the models made by SWISS-MODEL (Additional Fig. S2). As the position of R143 is located at a distance from the inner pore region where ion permeation occurs (even the nearest Ile 9 residue is at least 16.50 Å away), the R143W variant would have little direct effect from electrostatic interactions with potassium ions moving through the pore. Hence, the R143W variant, rather than directly affecting ion permeability, was predicted to destabilize the 3-D structure of each protomer (one unit of six), subsequently resulting in instability of the whole hemichannel (connexon) structure. This instability would also result in structural changes in the whole gap junction structure, consequently decreasing ion permeability indirectly. If the instability of the protomer with the R143W variant leads to loss of function, the level of hearing loss could be primarily determined by the level of residual function of the GJB2 protomer from the other allele. In this study, profound hearing loss was detected in patients with the p.[R143W];[R143W] genotype. Moreover, previous studies have also documented severe to profound hearing loss in all patients with the p.[R143W];[R143W] genotype [10, 11, 34]. Extremely reduced hearing levels in patients with compound heterozygotes of R143W with either a frameshift or a nonsense variant have also been observed. In case of patients with frameshift variants, patients with the p.[R143W];[G12Vfs*2 (c.35delG, rs80338939)] genotype showed profound hearing loss [5]. This finding on profound hearing loss is consistent with the prediction that the presence of the R143W variant results in severe hearing loss through the complete elimination of the gap junction function. Young patients with the p.[R143W];[L79Cfs*3] compound heterozygous variant often have profound hearing loss (75%), but relatively severe (12.5%) or moderate hearing loss (12.5%) is also observed (Additional Table S1). Childhood hearing impairment with *GJB2* variations involves all frequencies and is of variable severity [35]. The variation in hearing loss severity could not be satisfactorily explained only from the view point of structural biology. More age-specific hearing data from patients with the same variant will be needed to clarify the age-specific effects of p.[R143W];[L79Cfs*3] on hearing levels.

Unlike the R143W variant that showed structural instability, only minor structural and physical–chemical changes were observed in the V37I variant when compared with the wild type. Contrary to the findings in patients with the R143W homozygote or p.[R143W];[truncating variant] genotypes, our finding that none of the patients with the p.[R143W];[V37I] genotype showed profound hearing loss is consistent with the results of previous reports [31, 34–38]. These results imply that the V37I variant does not significantly affect the stability of the connexon structure. Further, no significant changes in the interactions (including hydrophobic interactions) with the surrounding residues were observed when valine 37 was substituted with isoleucine. Recently, a Chinese group showed that V37I does not affect connexon formation, but causes the aggregation of detached inner wall N-terminal “plugs” and reduces channel ion flow, as revealed by molecular dynamics (MD) simulations [39]. This report suggests that the V37I variant does not affect connexon formation, but is associated with hearing loss.

Therefore, it was predicted that the variant does not lead to a distinct decrease in structural function, resulting in relatively mild hearing loss in patients with the p.[R143W];[V37I] genotype. From the above findings, not only the distance from the ion pore but also the characteristics of the variants, including physical–chemical changes, should be considered when investigating the effect of these variants.

In in vitro studies, the R143W variant was localized in the region of cell–cell contact and may have formed functional gap junction channels with a value of conductance similar to that of wild-type GJB2 [7]. In addition, the coexpression of the wild-type GJB2 and N206S variant resulted in the formation of more stable channels compared with the expression of the N206S variant alone[40]. In vitro coexpression studies of the wild-type GJB2 and subjected variants (L90P, R127H, and R143W) that were injected into *Xenopus* oocytes exhibited significantly low levels of conductivity compared to that of the wild type[9]. These variants are located on the transmembrane domain of the protomer, far from the inner pore region, similar to R143W or N206S. They also lead to the formation of hemichannel structures in the aforementioned in vitro expression systems. These reports and our model suggest that the structural defect caused by the R143W variant allows gap junctions to form, but causes distortion of connexon structure and reduces ion permeability.

In future research, we will quantitatively investigate the energy differences between the wild type and variants by performing more detailed molecular dynamics calculations, such as by the free energy perturbation method. This analysis would enable quantification of the destabilization of the protomer structure caused by R143W, and is expected to clarify the correlation between structural distortion and hearing level.

## Limitations

The findings obtained in this study indicate that the R143W variant associated with the loss of a hydrogen bond may form an expressible hemichannel structure as a protein, which is unstable and functionally abnormal. However, more cases of patients with the R143W variant will be needed to prove our molecular pathology model, which can explain patients’ hearing level.

## Conclusion

We concluded that the R143W variant, which causes structural destabilization in the structural model of GJB2, provides interpretation of the severity of the molecular pathology and can serve as an alternative to clinical data. Accumulation of structural models, in vitro experimental data, and careful evaluation of clinical data focused on genotype–phenotype correlation would provide a precise understanding of the molecular pathology of *GJB2* variants. However, in order to make these molecular pathologies more authoritative, cases of patients with the same variant are needed.

### Supplementary Information

**Additional file 1: Fig. S1.** Comparison of the hydrophobic environment between the wild type and V37I variant.

**Additional file 2: Fig. S2.** Comparison between wild type crystal structure (gold) and variant models (R143W (cyan), N206S (light green) and N206T (light gray)) predicted by AlphaFold2.

**Additional file 3: Table S1:** Clinical data of patients with each genotype.

## Data Availability

Data of hearing level of 22 patients who appeared to have two *GJB2* pathogenic variants are available at Division of Hearing and Balance Research, National Institute of Sensory Organs.
