# Supplementary figures and images for: Structural basis for pathogenic variants of GJB2 and hearing levels of patients with hearing loss
Source: BMC Res Notes. 2024 May 10;17:131. doi: 10.1186/s13104-024-06793-w (PMC11083831; doi:10.1186/s13104-024-06793-w)

Additional figure 1

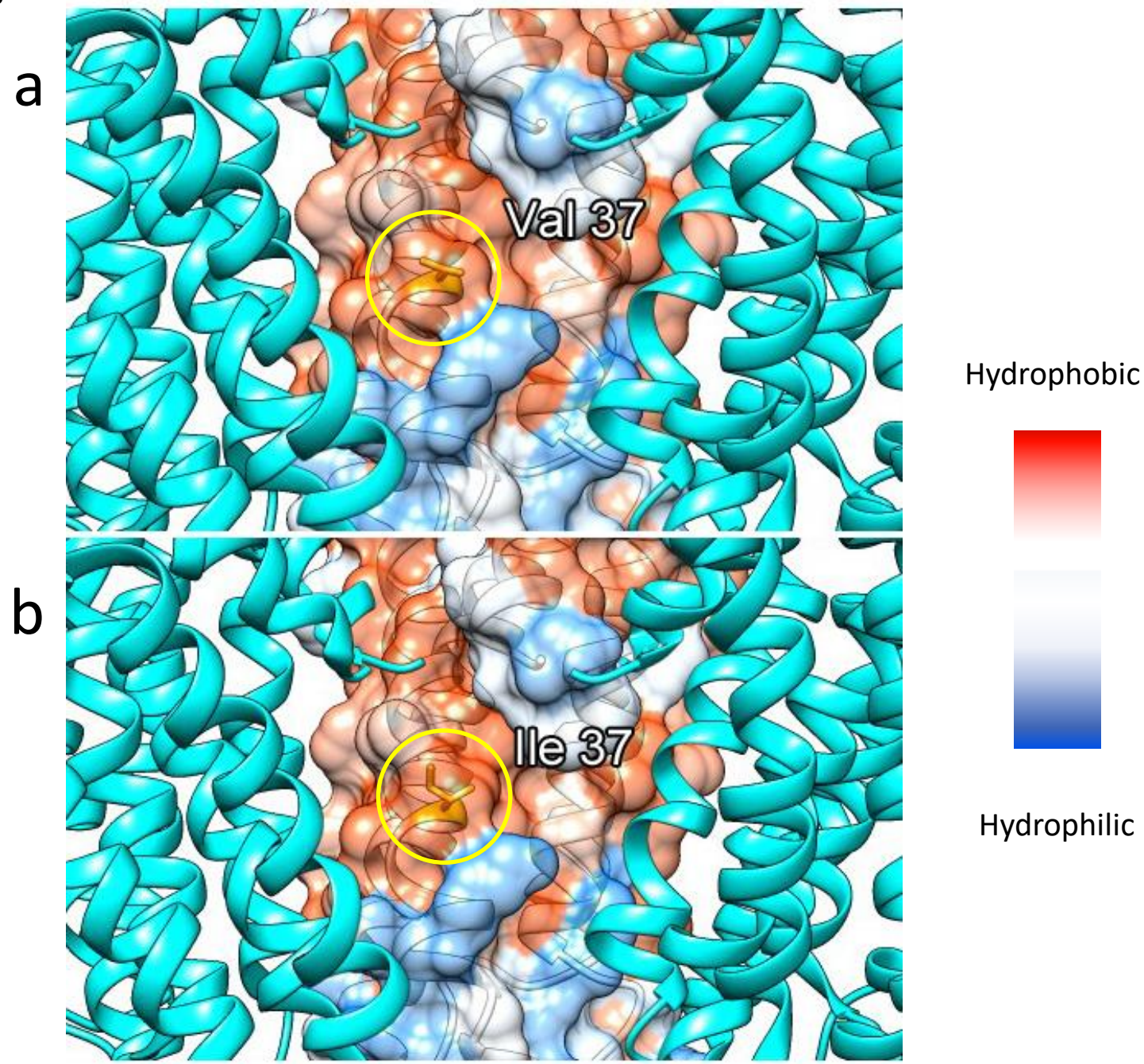

Supplement: Supplementary file 1 — Additional file 1: Fig. S1. Comparison of the hydrophobic environment between the wild type and V37I variant. [file 13104_2024_6793_MOESM1_ESM.pdf]

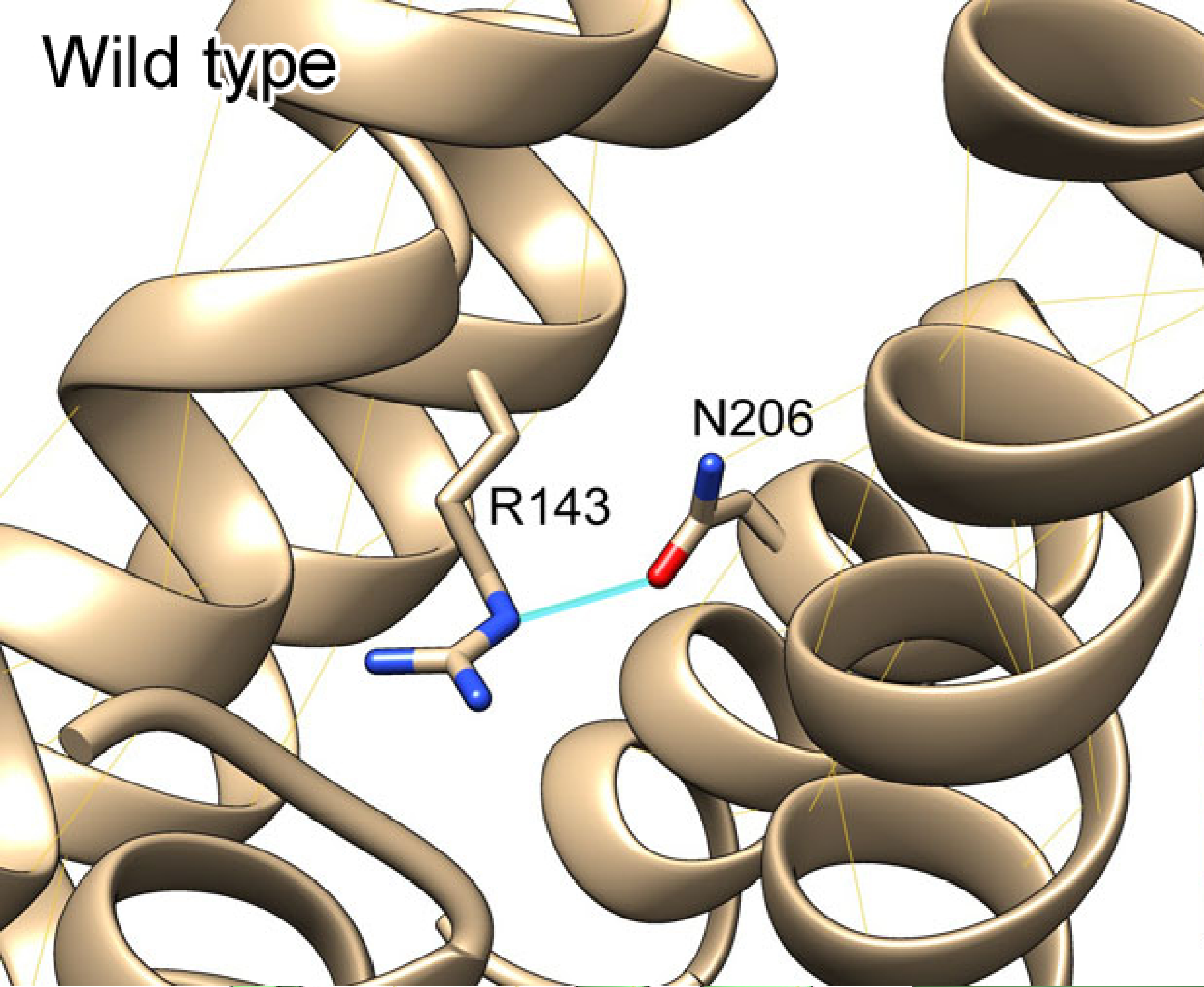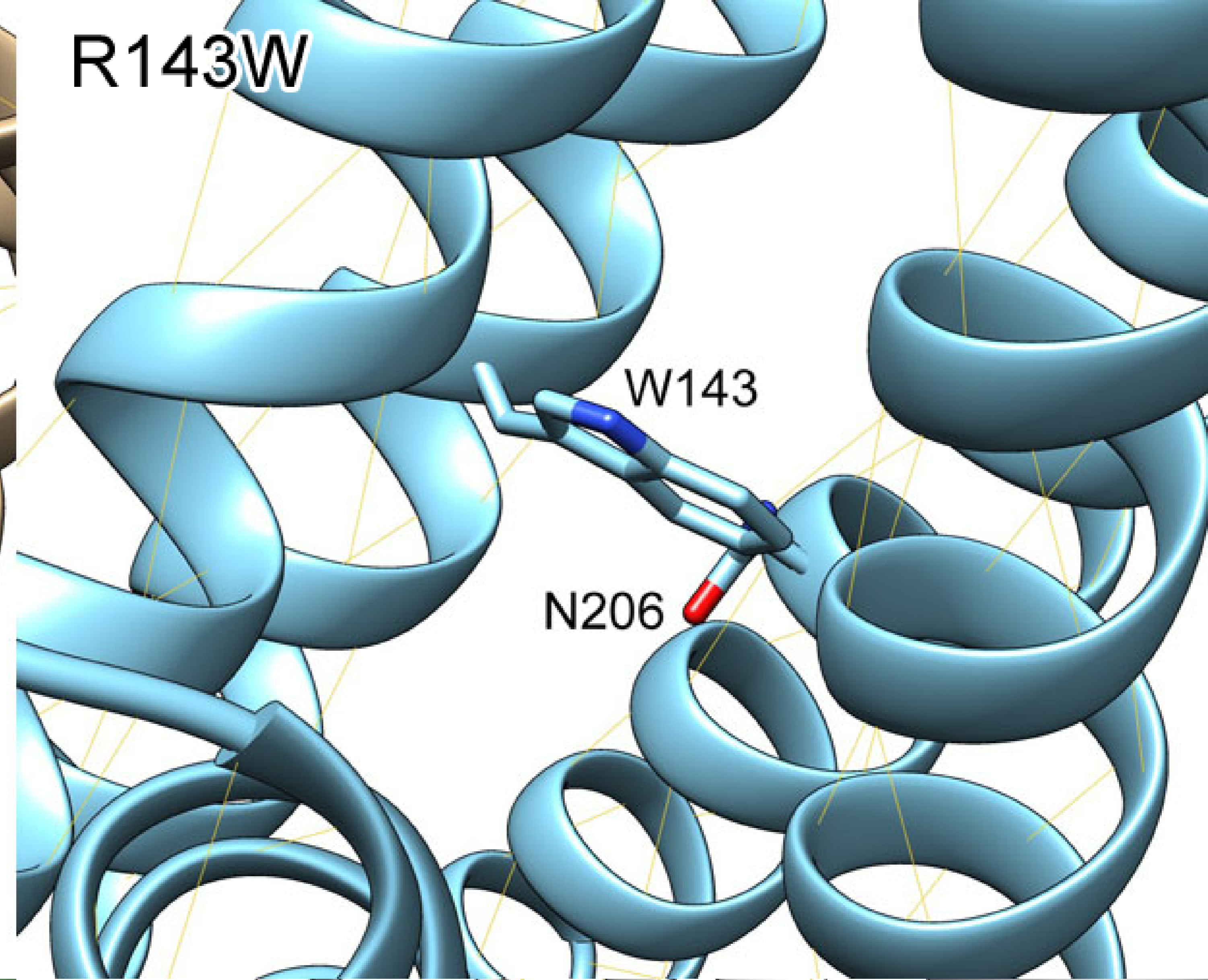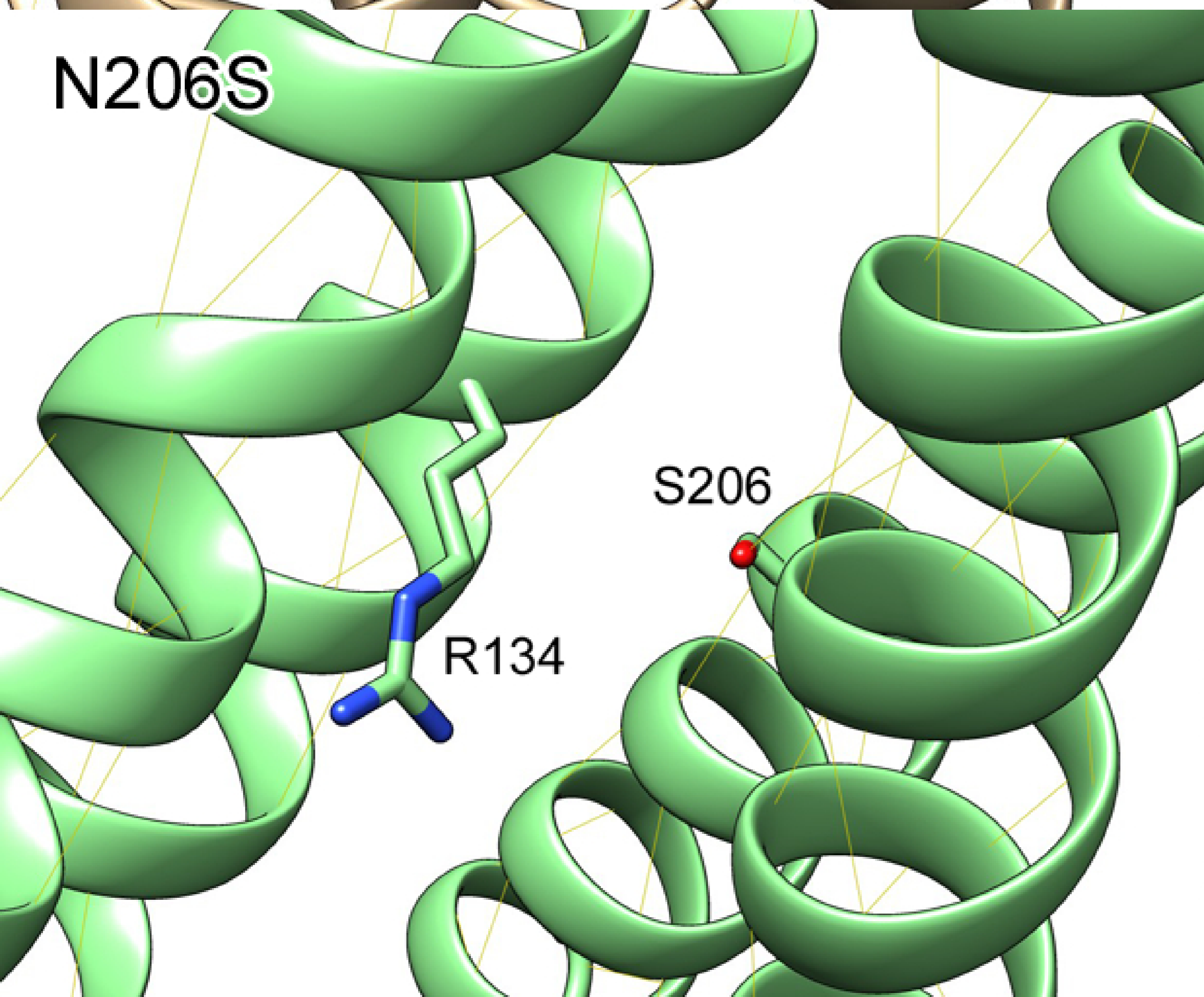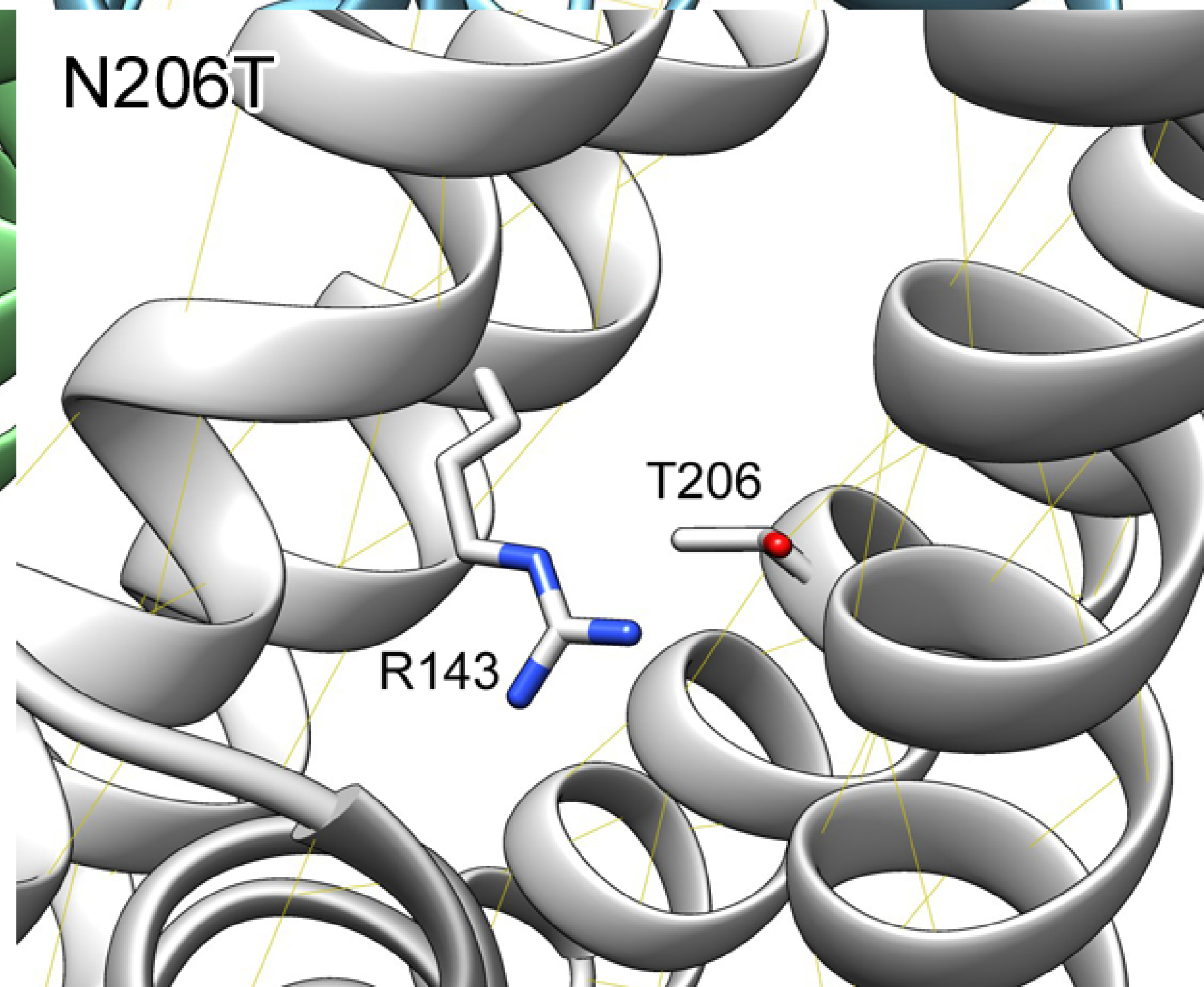

Supplement: Supplementary file 2 — Additional file 2: Fig. S2. Comparison between wild type crystal structure (gold) and variant models (R143W (cyan), N206S (light green) and N206T (light gray)) predicted by AlphaFold2. [file 13104_2024_6793_MOESM2_ESM.pdf]
