# Supplementary material for: Structural basis for pathogenic variants of GJB2 and hearing levels of patients with hearing loss
Source: BMC Res Notes. 2024 May 10;17:131. doi: 10.1186/s13104-024-06793-w (PMC11083831; doi:10.1186/s13104-024-06793-w)
Supplement: Supplementary file 3 — Additional file 3: Table S1: Clinical data of patients with each genotype. [file 13104_2024_6793_MOESM3_ESM.pdf]

Additional Table S1 Clinical data of patients with each genotype

Genotype	Age (year)	Sex	Progression	Reference
p.[R143W];[V37I]	23	male	Yes	This study
	1	male	Yes	
	4	male	No	
	7	female	Yes	
	19	female	No	
	20	male	Yes	
	29	female	No	
	30	male	Yes	
	34	female	Yes	
	49	male	Yes	
	4	female	No	
p.[N206S];[V37I]	not available	female	Unknown	[13]
p.[N206S];[35delG]	not available	Female	Unknown	[24]
	not available	Female	Unknown	
p.[N206T];[W24X]	not available	not available	Unknown	[14]
p.[R143W];[H73Y]	11	male	No	This study
p.[R143W];[R143W]	1	male	No	This study
p.[R143W];[H100Rfs*14]	1	male	No	This study
p.[R143W];[L79Cfs*3]	0	male	Unknown	This study
	4	female	No	
	0	male	No	
	0	male	No	
	0	female	Unknown	
	0	male	No	
	0	male	No	
	0	female	No	
	0	female	No	
	1	female	No	
	1	female	No	
	1	female	Unknown	
	2	female	No	
	3	male	No	
	3	male	Yes	
	37	male	Yes	
p.[R143W];[G45E;Y136*]	0	male	No	This study
	5	female	Unknown	
p.[R143W];[A171Efs*40]	0	female	No	This study
	2	female	Yes	

Mild Moderate Severe Profound
